# Lactoperoxidase: Properties, Functions, and Potential Applications

**DOI:** 10.3390/ijms26115055

**Published:** 2025-05-24

**Authors:** Hasan Kutluay Özhan, Hatice Duman, Mikhael Bechelany, Sercan Karav

**Affiliations:** 1 Department of Molecular Biology and Genetics, Çanakkale Onsekiz Mart University, Çanakkale 17100, Türkiye; ozhan.hasankutluay@gmail.com (H.K.Ö.); hatice.duman@comu.edu.tr (H.D.); 2 Institut Européen des Membranes (IEM), UMR 5635, University Montpellier, ENSCM, CNRS, F-34095 Montpellier, France; 3 Functional Materials Group, Gulf University for Science and Technology (GUST), Masjid Al Aqsa Street, Mubarak Al-Abdullah 32093, Kuwait

**Keywords:** lactoperoxidase, milk, antibacterial, antiviral, antifungal, enzyme, oxidation, therapeutic

## Abstract

Lactoperoxidase (LPO) (E.C. 1.11.1.7) is a member of the superfamily of mammalian heme peroxidases that is isolated from milk, and it is the first enzyme announced to be found in milk. In addition to milk, LPO is also found in saliva, tears, and airways (airway goblet cells and submucosal glands). It contributes significantly to the self-defense of the mammal body. It catalyzes the oxidation of certain molecules such as thiocyanate (SCN^−^), I^−^, and Br^−^ in the presence of hydrogen peroxide (H_2_O_2_). This reaction leads to the formation of antimicrobial products that have a great antimicrobial spectrum, including antibacterial, antiviral, and antifungal activity, especially hypothiocyanite (OSCN^−^) and hypoiodite (OI^−^), which are coming into prominence via their high antimicrobial activity. The lactoperoxidase system (LPOS) is the system consisting of LPO, H_2_O_2_, and SCN^−^. LPO has a great potential to be used in various areas such as preservation and shelf-life elongation of milk; milk products; meat; meat products; plants, including fruits and vegetables; and oral care, diagnosis, immunomodulation, and treatment of nephrotoxicity. The LPO gene, along with LPO itself, is important for animals. In the absence of the LPO gene, there is an increase in the frequency of diverse diseases, including inflammation, tumor formation, and obesity. In this review, we mentioned general information about the enzyme LPO and its potential. Chemical properties and other features of other components of the LPOS, H_2_O_2_, and SCN^−^ were also touched on the review. To offer readers a comprehensive understanding of the enzyme’s biological significance and research progress over time, both recent and older studies have been used together. Lastly, we discussed potential applications of LPO in different areas and left future remarks in the light of recent studies.

## 1. Introduction

Peroxidases (E.C 1.11.1.X) catalyze the decomposition of a range of peroxides (ROOH), typically H_2_O_2_, facilitating the oxidation of both organic and inorganic substrates [1]. Lactoperoxidase (LPO) (E.C. 1.11.1.7) is the name given to the peroxidase enzyme obtained from milk [2,3,4,5], and LPO is the first enzyme announced to be found in milk [6]. LPO is a kind of heme-containing peroxidase (HPO) enzyme, it is found in saliva, milk, and tears. Additionally, it is also found in airways. In sheep, it has been demonstrated that the peroxidase activity present in airway goblet cells and submucosal glands is attributable to LPO [7]. This enzyme has an important biological role as a part of the immune system; it catalyzes the oxidation of certain molecules [3,4,8,9], thus providing antibacterial [2,3,4,8,9,10,11,12,13,14,15,16,17,18,19,20], antiviral [3,5,8,9,21,22,23], and antifungal [3,24,25] properties to LPO. Furthermore, a bacterial form of LPO, named CyanoPOX, was recently discovered and characterized. CyanoPOX shows 37% sequence identity with bovine LPO, and it was found in *Cyanobacterium* sp. TDX16. Although its identity is relatively low, it shows some similarities with mammalian LPOs, such as pH stability. Additionally, it was successfully overexpressed in *Escherichia coli* [26].

LPO works with hydrogen peroxide (H_2_O_2_), thiocyanate (SCN^−^), I^−^, and Br^−^. Although it can catalyze the oxidation of some halides such as I^−^ and Br^−^, it cannot catalyze the oxidation of Cl^−^ [6,27]. The system that consists of LPO/H_2_O_2_/SCN^−^ was called the lactoperoxidase system (LPOS) [6,27]. In this system, the oxidation of SCN^−^ is catalyzed by LPO in the presence of H_2_O_2_ to yield hypothiocyanite (OSCN^−^), which exhibits antimicrobial actions [4,5,6,27]. OSCN^−^ interacts with sulfhydryl (-SH) groups of proteins, thereby inhibiting important metabolic pathways—particularly enzymes involved in glycolysis and glucose transport systems, resulting in a bacteriostatic effect [4,6,28]. Short-lived intermediary oxidation products of SCN^−^, which were formed by the reaction that was catalyzed by LPO, may be reduced back into SCN^-^ or they can undergo further oxidation to form end products, such as ammonia, carbon dioxide, and sulfate. The primary intermediary oxidation product of the reaction is OSCN^−^; moreover, some kinetic and polarographic studies have indicated that very short-lived oxidation products, such as O_2_SCN^−^ and O_3_SCN^−^, may be produced when H_2_O_2_ is present in concentrations exceeding those of SCN^−^ [2].

SCN^−^ anions are found in many places of the mammal body, such as in tissues and secretions such as salivary, mammary, and thyroid glands and their secretions; stomach and kidneys; synovial, cerebral cervical and spinal fluids; lymph; and plasma [27].

The LPOS provides antibacterial properties to milk; OSCN^−^ ions are short-lived components, and the nascency of them exhibits bacteriostatic effect [3,4,6,9]. The components of the LPOS are naturally found in milk and the system continues its bacteriostatic activity for at least 1 h after milking [11]. LPO protects the intestinal tract and lactating mammary gland of the newborn infants against harmful microorganisms. The most common method used for preventing the deterioration of milk is cooling, but this method is not readily available for many people [3]. The system is promising on the path of the storage and transportation of milk and milk products [3,12,15,17,25,29].

LPO presents in the whey fraction of milk, and 0.25–0.5 percent of total protein found in milk consists of LPO [29]. In bovine milk, LPO occupies approximately 0.5% of protein in whey; the concentration of LPO is around 30 mg/L, and this situation makes it the most abundant enzyme next to xanthine oxidase. In bovine colostrum, LPO concentration increases for a time after postpartum, reaching its maximum level after 3–5 days postpartum, then decreases slowly and reaches a plateau after about 2 weeks [27]. The LPO content of bovine colostrum is 11–45 mg/L, and the catalase activity of the LPO is higher in bovine colostrum than in mature milk [9,30].

LPO contributes significantly to the immune defense of mammals against pathogens due to its antibacterial [2,3,4,8,9,10,11,12,13,14,15,16,17,18,19,20], antiviral [3,5,8,9,21,22,23,31], and antifungal [3,24,25] activity. In addition to the potential of LPO in the storage and transportation of milk and milk products [11,13,16], scientists know that LPO has great potential in various areas, such as the preservation of meat and meat products [12,15,17], preservation of fruits [25], oral health [14,18,19], and even diagnosis of diseases [32,33]. This indicates that it is promising at a point, providing very influential benefits for human life. At this point, the identification of LPO inhibitors becomes important; there are plenty of known inhibitors for LPO.

There are several types of LPO inhibitors, including drug substances such as 2,6-Dimethylphenol, 2,6-di-T-butylphenol, di(2,6-dimethylphenol), di(2,6-diisopropylphenol), di(2,6-di-T-butylphenol) [34], ketamine, and bupivacaine [35]; vitamins such as L-Ascorbic acid (vitamin Q), Menadione sodium bisulfate (vitamin K3), and folic acid (vitamin B9) [36]; hormones, like L-adrenaline [37] and norepinephrine [38]; and anthraquinone, 1,2-dihydroxy-anthraquinone, 1,5-dihydroxy-anthraquinone, 2,6-dihydroxy-anthraquinone, 1,8-dihydroxy-3-methyl-anthraquinone, 1,4-dihydroxy-2,3-dimethyl-anthraquinone [39]. Additionally, in the presence of riboflavin (vitamin B2), LPO becomes very sensitive to light and can undergo photochemical inactivation. For example, when milk, whey, and permeate were exposed to 6000 lux for 4 h at 10 °C, LPO was inactivated by 55%, 75%, and 97%, respectively. Although the photochemical inactivation was irreversible, it could be nearly completely prevented by adding cysteine. On the other hand, in a buffer containing 50 ppm LPO at 6.7 pH, photochemical inactivation of LPO was not observed when riboflavin was not added [27].

There are various chromatographic techniques known about LPO purification and its characterization from milk [40]. Some ways to remove LPO from milk are CM-Cellulose [41], CM-Sephadex ion-exchange chromatography [41,42], Sephadex G-100 gel filtration chromatography [41,43], hydrophobic affinity chromatography on Phenyl-Sepharose CL-4B [44], and Toyopearl-SP cation exchange chromatography [41]. Additionally, the affinity technique can purify LPO in one stage, using sulfanilamide as a ligand [45].

In summary, LPO is a crucial enzyme for mammals due to its high antibacterial, antifungal, and antiviral activity; it can protect newborn infants from harmful microorganisms during lactation. It can also protect tissues from the harmful effects of H_2_O_2_. In addition to its importance for the mammal body, it has great potential to be used as a beneficial material in various areas, such as the food industry, medicine, agriculture, dairy industry, and veterinary medicine. This comprehensive review aims to provide an in-depth overview of LPO, incorporating both the classical and recent literature to illustrate the enzyme’s long-standing presence and continued relevance in scientific research. By integrating historical findings with up-to-date studies, the review highlights the evolution of knowledge surrounding LPO and its biological significance. In addition to offering a general introduction to the LPOS, this article compiles recent research efforts on the enzyme, shedding light on its biochemical characteristics and physiological roles. As an original contribution, the review also discusses and interprets the potential applications of LPO in various fields, emphasizing its promising utility in biomedical, pharmaceutical, and industrial contexts. Future studies should investigate the antimicrobial activity of the LPO strain specifically to understand the wide antimicrobial spectrum of LPO in more detail, including determining dosage at specific conditions. However, it has to be more studied to develop treatments against particular diseases and to use in industrial areas, such as the preservation of foods.

## 2. Components of LPOS

### 2.1. Lactoperoxidase

LPO is classified under the superfamily of mammalian heme peroxidases; this family involves myeloperoxidase (MPO), eosinophil peroxidase (EPO), thyroid peroxidase (TPO), and LPO [46]. All these enzymes exhibit similar functions and have similar structures, particularly on active sites [47]. The LPO enzyme obtained from bovine is composed of a single polypeptide chain; it contains 612 amino acid residues with known sequences [27], including 15 cysteine residues [48]. The molecular weight of the molecule is approximately 78 kDa. The isoelectric point of bovine LPO is 9.6. Around 10% of an LPO molecule consists of carbohydrates, and it has five potential *N*-glycosylation sites. Each LPO molecule contains a calcium ion bonded strongly; the activity of this calcium ion is crucial to the structural integrity of the LPO molecules, as it stabilizes the conformation of the molecule [27]. The calcium atom of an LPO molecule is linked to Asp227, as shown in numerous peroxidases. If an LPO molecule forfeits its calcium-binding ability, it entirely loses its functionality [48]. There are at least 10 different LPO fractions that have been identified [49]. Each LPO molecule contains one iron atom as a part of a heme group, and this iron content forms 0.07% mass of an LPO molecule. Different LPO fractions do not exhibit significantly different enzymatic activity compared to other fractions. Each LPO molecule has one heme group as protoheme 9 [50]. The catalytic center of the LPO molecule contains the heme group, known as protoporphyrin IX. Also, this heme group is attached covalently to the polypeptide chain via an ester bond [51].

As mentioned earlier, LPO is widely distributed across several biological fluids [52]. Different species vary in the concentration of LPO and its enzymatic activity [6] as shown in the Table 1. The LPO content of bovine milk is about 30 mg/L [6,53], and human whey contains 0.77 ± 0.38 mg/L LPO [43]. Cow milk has 20 times more peroxidase activity than human milk [6].

Human LPO and bovine LPO show similarity from many sides; their differences between their physicochemical properties, chemical activity, and structure are small. Both bovine LPO and human LPO are single chains. According to mRNA studies, both bovine LPO and human LPO contain 712 amino acid residues, while according to protein analysis, human LPO contains 632 amino acid residues, including 16 cysteine residues, and bovine LPO contains 612 amino acid residues, including 15 cysteine residues. The difference between the theoretical length of the chain and the length of the analyzed protein comes from the post-translational modifications, the cleavage of the propeptide, and the signal peptide. The molecular mass of human LPO is 80 kDa, and it has four potential *N*-glycosylation sites [48].

In humans, the LPO gene is found on chromosome 17, while the bovine LPO gene is found on chromosome 19. The same gene produces human LPO in saliva and in other body fluids [48]. The human LPO gene has 28 kb long, and it contains 12 exons and 11 introns. The gene also includes a 1.5 kb region located 5u upstream. The human LPO gene’s coding sequence can encode a protein consisting of 712 amino acids; the sequence has 2136 nucleotides. Bovine LPO and human LPO are 83% similar at the amino acid level and 86% similar at the nucleotide level [59]. The mRNA of human LPO can undergo alternative splicing; in this way, three possible transcript variants (V1, V2, and V3) are formed. V1 includes a complete coding sequence, V2 lacks exon 3, and V3 lacks both exon 3 and 4. V1 and V3 have enzymatic activity, and the enzymatic activity of V3 is higher than that of V1 [60].

LPO’s three-dimensional structure is mostly composed of alpha helices and beta sheets. When LPO is in an active state, the heme moiety is entirely attached to the protein portion, and two covalent linkages are established through Asp and Glu residues; when LPO is in an inactive state, there is a partial linkage between the heme moiety and the protein part, and one covalent linkage is established through the Asp residue [61].

Studies with buffer, permeate, whey, and milk indicated that the denaturation of LPO due to heat starts at about 70 °C. The thermal stability of LPO in permeate and buffer was lower than that of milk or whey. Additionally, the concentration of calcium ions was strongly effective on the heat sensitivity of LPO. The stability of LPO decreases in an acidic environment (pH 5.3), likely because of calcium ions being released from the LPO molecule [27]. LPO is considered among the most thermally stable enzymes present in milk [49,62,63]; it can undergo partial inactivation by short-time pasteurization at 74 °C, and after this, it still shows adequate activity to catalyze the conversion of SCN^−^ to OSCN^−^ when H_2_O_2_ is present [63]. We can use the destruction of LPO as an indicator of milk pasteurization efficiency [62]. During the standard pasteurization of cow’s milk (at 63 °C for 30 min or at 72 °C for 15 s), LPO maintains its activity. LPO is destroyed when exposed to 80 °C for 2.5 s and completely inactivated in 15 s at 78 °C [3]. In 2001, a study indicated that LPO is not inactivated by normal pasteurization of milk. The LPOS worked against *Pseudomonas aeruginosa*, *Staphylococcus aureus*, and *Streptococcus thermophilus* in milk that had these bacteria added to it [3,64]. The milk had been pasteurized at 72 °C for 15 s. LPO becomes thermally inactivated when the temperature is close to that at which the native structure of the LPO unfolds and follows first-order kinetics [27].

### 2.2. Thiocyanate (SCN^−^)

Thiocyanate (SCN^−^) is a small, highly acidic pseudohalide thiolate that is widely distributed in the extracellular fluids of mammals. It contributes to host defense as a component of the LPO-mediated antimicrobial system. It serves as the preferred substrate for LPO-catalyzed reduction of H_2_O_2_, resulting in the formation of hypothiocyanous acid (HOSCN). HOSCN is selectively produced by several peroxidase enzymes capable of utilizing SCN^−^, including eosinophil peroxidase (EPO), gastric peroxidase (GPO), myeloperoxidase (MPO), salivary peroxidase (SPO), and thyroid peroxidase (TPO). These enzymes facilitate HOSCN generation through a two-electron halogenation reaction. SCN^−^ is present in various biological secretions—including plasma, saliva, airway epithelial lining fluid, nasal lining fluid, milk, tears, and gastric juice–across a broad concentration range (0.01–3 mM) [65] as shown in the Table 2. It is detected in glands such as salivary, mammary, and thyroid glands as well as their respective secretions. Moreover, SCN^−^ is observed in organs such as the stomach and kidneys as well as in bodily fluids such as cerebral fluid, synovial fluid, cervical fluid, spinal fluid, and lymph [2]. In bovine milk, the concentration of SCN^−^ ions depend on some factors such as the breed, species, udder health, and type of feed. Also, SCN^−^ concentration in bovine milk reflects blood serum levels. Additionally, colostrum contains more SCN^−^ than that in milk [27]. In bovine milk, different SCN^−^ levels are normally between 1 ppm and 10 ppm, but higher values have been reported too, especially in milk with a high somatic cell count [2]. In a publishment, 10 mg of SCN^−^ per liter of fresh bovine milk was reported, and this amount is not always sufficient to activate LPOS [66]. Other reports showed that the SCN^−^ concentration in human saliva is 50–300 ppm [67] and in human gastric juice 40–50 ppm [68]. These values significantly exceed the required SCN^−^ concentration (15 ppm) to activate LPOS and its bactericidal activity [67]. In the human body, the concentration of SCN^−^ can change by smoking; human body fluids of smokers contain more SCN^−^ than non-smokers. SCN^−^ is excreted with urine, and its excretion rate increases with increasing serum concentration; the half-life of the elimination takes 2–5 days when renal functions are normal [2].

SCN^−^ is introduced into the human body through dietary sources, particularly cruciferous vegetables, or is endogenously produced from cyanide via the action of sulfurtransferase enzymes, such as mitochondrial rhodanese and cytosolic mercaptopyruvate sulfurtransferase [65]. The primary dietary sources of SCN^−^ are glucosinolates and cyanogenic glucosides [63]. The hydrolysis of glucosinolates yields SCN^−^ and some other products. Some vegetables are rich in glucosinolates, for example, kale, cabbage, brussel sprouts, cauliflower, rutabaga, and turnips. When cyanogenic glucosides are hydrolyzed, cyanide is released by them, and the cyanide is in a reaction with thiosulphate (a metabolic product of sulphur-containing amino acids) and is detoxified through its conversion into SCN^−^. The latter reaction is catalyzed by the enzyme rhodanese (EC 2.8.1.1). This is also how the metabolism of cyanide from tobacco smoke occurs. Maize, millet, cassava, sweet potatoes, sugar cane, peas, beans, and the kernel of some fruits are among the sources of cyanogenic glucosides [2].

### 2.3. Hydrogen Peroxide (H_2_O_2_)

A covalent liquid with pale-blue color, hydrogen peroxide (H_2_O_2_) is easily miscible with water, and it easily passes through cell membranes [79]. Due to its low reactivity, H_2_O_2_ can serve as either a weak oxidizing agent or a weak reducing agent. The majority of biomolecules, such as DNA, lipids, and proteins, are not oxidized by H_2_O_2_. High levels (usually ≥50 µM) of H_2_O_2_ are cytotoxic for many animals, plants, and bacterial cells in culture. The cell type, physiological state, exposure time to H_2_O_2_, concentration of H_2_O_2_, and cell culture medium used can affect LD_50_ values and the mode of cell death induced (apoptosis or necrosis). H_2_O_2_ is highly toxic in vivo; for this reason, its quick elimination is very important. Its elimination can be accomplished through enzymes like catalases, peroxidases (especially the glutathione peroxidase enzymes), and thioredoxin-linked systems. The harmful effect of H_2_O_2_ is mainly caused by its quick transformation to reactive hydroxyl radical, and this conversion can occur because of UV light exposure or through interactions with certain transition metal ions, such as iron [80]. H_2_O_2_ can be produced by lactic acid bacteria via their metabolic activities; sufficient H_2_O_2_ molecule can be produced by many lactobacilli and streptococci under aerobic conditions for the activation of the LPOS. Also, investigations showed that H_2_O_2_-producing bacteria are present in human saliva [2].

## 3. Biological Functions of LPO

**Figure 1:**
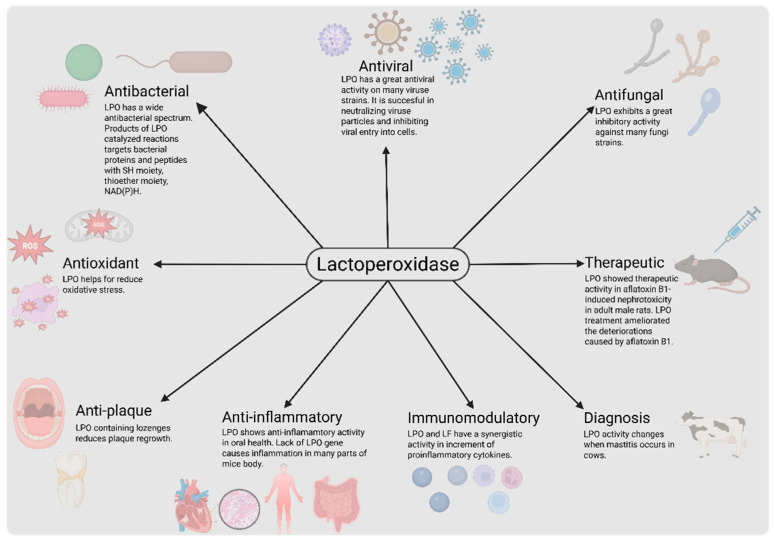
Known functions of LPO [10,11,12,13,14,15,16,17,18,19,20,21,22,23,24,25,32,81,82,83,84,85,86,87].

The LPO system is a multi-functional system, and it has important roles in the continuity of life (Figure 1). The system is promising for use in different areas in modern human life via its antibacterial [10,11,12,13,14,15,16,17,18,19,20,81], antifungal [24,25,81,82,83], antiviral [21,22,23,84,85], antiplaque [18,19], anti-inflammatory [19,86], immunomodulatory [20], antioxidant and therapeutic [87], antitumor [86], and health indicator [32] properties. There are a lot of studies in the literature about these properties, and new usage areas are found in time (Table 3).

### 3.1. Antibacterial Properties of LPO

**Figure 2:**
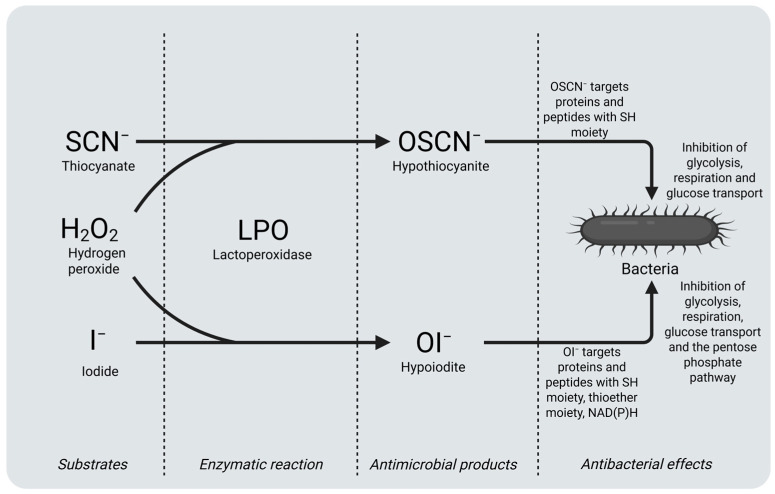
Production of OSCN^−^ and OI^−^ by LPO catalyzed reactions [6,27] and their antibacterial activity [28].

Bacterial enzymes such as hexokinase and glyceraldehyde-3-phosphate dehydrogenase (GAPDH) have -SH groups, and LPOS causes oxidation of these sulfhydryl (thiol) groups; thus, the enzymes lose their biological functions. Consequently, the uptake of glucose, purine, pyrimidine, and amino acids and the synthesis of protein, DNA, and RNA are blocked. All these properties cause prevention of bacterial growth and bacterial proliferation [6]. The sulfhydryl group plays a critical role in the functionality of various enzymes and proteins. The oxidation of key glycolytic enzymes—such as hexokinase, GAPDH, aldolase, and glucose-6-phosphate dehydrogenase—has been shown to inhibit bacterial glycolysis. Additionally, disruptions in cellular respiration and glucose transport are linked to modifications of the cell membrane or associated transport proteins. OSCN^−^ targets the thiol moiety (-SH groups) of proteins and peptides to oxidize them. This stops bacteria from breaking down sugar, breathing, and transporting glucose (Figure 2). In addition to SCN^−^, LPO also can catalyze the oxidation of I^−^ to OI^−^, which targets the oxidization of the SH moiety, thioether moiety, or NAD(P)H of peptides and proteins, thus inhibiting glycolysis, respiration, glucose transport, and the pentose phosphate pathway of bacteria [28]. In a study based on the LPO-iodide-hydrogen peroxide combination, the sensitivity of different *Actinobacillus actinomycetemcomitans* strains to the combination is compared with sensitivities to different antibiotics. *Actinobacillus actinomycetemcomitans* is found in periodontal pockets and whole saliva; this microorganism plays an important etiological role in localized juvenile periodontitis and progressive periodontitis. The study showed that a combination that consists of LPO (75 micrograms), I^−^ (100 nmol), and H_2_O_2_ (1000 nmol) exhibited an inhibition on *Actinobacillus actinomycetemcomitans* similar to 2 micrograms of ampicillin [10].

In a study, the antibacterial activity of LPOS consisted of goat milk LPO, SCN^−^, and H_2_O_2_ was tested on strains of *Aeromonas hydrophila*, *Citrobacter freundi*, *Escherichia coli*, *Klebsiella pneumoniae, Proteus mirabilis, Pseudomonas aeruginosa, Salmonella enteritidis bioser Paratyphi A, Salmonella schotmuelleri, Salmonella typhi, Serratia marcescens, Shigella dysentriae, Shigella sonnei, Staphylococcus aureus, Staphylococcus citreus,* and *Vibrio cholerae* using disc diffusion method. The system was effective on all tested strains. The inhibition zone of *Aeromonas hydrophila* was 22 mm, of *Citrobacter freundi* was 20 mm, of *Escherichia coli* was 22 mm, of *Klebsiella pneumoniae* was 23 mm, of *Proteus mirabilis* was 24 mm, of *Pseudomonas aeruginosa* was 20 mm, of *Salmonella enteritidis* bioser Paratyphi A was 22 mm, of *Salmonella schotmuelleri* was 25 mm, of *Salmonella typhi* was 23 mm, of *Serratia marcescens* was 24 mm, of *Shigella dysentriae* was 26 mm, of *Shigella sonnei* was 26 mm, of *Staphylococcus aureus* 24 mm, of *Staphylococcus citreus* was 23 mm, and of *Vibrio cholerae* was 26mm. The MIC values of goat LPO in the system for various strains were 176 µg/mL for *Aeromonas hydrophila*, 297 µg/mL for *Citrobacter freundi*, 158 µg/mL for *Escherichia coli*, 126 µg/mL for *Klebsiella pneumoniae*, 196 µg/mL for *Proteus mirabilis*, 297 µg/mL for *Pseudomonas aeruginosa*, 100 µg/mL for *Salmonella enteritidis* bioser Paratyphi A, 100 µg/mL for *Salmonella schotmuelleri*, 195 µg/mL for *Salmonella typhi*, 84 µg/mL for *Serratia marcescens*, 50 µg/mL for *Shigella dysentriae*, 112 µg/mL for *Shigella sonnei*, 182 µg/mL for *Staphylococcus aureus*, 182 µg/mL for *Staphylococcus citreus*, and 49 µg/mL for *Vibrio cholerae* [81].

In a study, the effects of camel LPO and camel LF against multidrug-resistant *Acinetobacter baumannii* were tested in vitro and in vivo. In the in vitro part of the study, the antibacterial activity of the purified LPO and purified LF on *Acinetobacter baumannii* isolates was tested by the disk diffusion method at 8, 16, 32, and 64 μg/mL concentrations, and the results of the study showed that there is significant antibacterial activity against all isolates at all concentrations compared to the control. Additionally, LPO exhibited significantly higher antibacterial activity than LF in the same conditions. In the in vivo part of the study, the antibacterial effects of imipenem, purified LF, purified LPO, and a combination of LF and LPO were tested in the lungs of mice and blood culture. LF, LPO, and LPO + LF treatments were applied on mice by injection. Significant antibacterial activity was observed in imipenem, LPO, LF, and LPO-LF combination samples in lung and blood culture compared to the control group. Comparison CFU measurements of the samples were imipenem > LF > LPO > LPO + LF in lung and imipenem > LF > LPO = LF + LPO in blood culture. In blood cultures, while LPO and LF + LPO samples inhibited the bacterial growth entirely and LF samples inhibited it almost entirely, imipenem could not act the same way, and remarkable bacterial activity was reported in the imipenem samples. In the study, the immunomodulatory effects of LPO and LF were also studied. In this part of the study, proinflammatory cytokines IL-4 and IL-10 levels in the lung were determined on the seventh day of infection by *A. baumannii*, and the results showed that a significant increment was observed in IL-4 and IL-10 levels of the mice in groups that were treated with LPO, LF, and LPO + LF; the increment was significantly lower in the imipenem-treated group. The highest increment was observed in the LPO + LF-treated group contrasted with the control group. This situation indicated that the crude combination of LPO and LF may be used for the treatment of pneumonia caused by *A. baumannii* in place of imipenem. To sum up, in vitro and in vivo studies showed that camel milk-sourced LPO, LF, and the combination exhibited a significant bacterial growth inhibition on *A. baumannii*. Also, LPO and LF have a high synergistic potential in the increment of proinflammatory cytokines IL-4 and IL-10. Eventually, the study showed that the camel LPO and LF have the potential to reduce the amount of multidrug-resistant *A. baumannii* in the lung and prevent death [20].

Studies clearly showed that LPO has a great antibacterial activity against many bacterial strains [10,11,12,13,14,15,16,17,18,19,20], such as *Actinobacillus actinomycetemcomitans* [10], *Listeria innocua*, *Pseudomonas fluorescens*, *Staphylococcus saprophyticus* [12], *Streptococcus mutans* [14,18], *Acinetobacter baumannii* [20], *Aeromonas hydrophila*, *Citrobacter freundi*, *Escherichia coli*, *Klebsiella pneumoniae*, *Proteus mirabilis*, *Pseudomonas aeruginosa*, *Salmonella enteritidis* bioser Paratyphi A, *Salmonella schotmuelleri*, *Salmonella typhi*, *Serratia marcescens*, *Shigella dysentriae*, *Shigella sonnei*, *Staphylococcus aureus*, *Staphylococcus citreus*, and *Vibrio cholerae* [81]. The potential of LPO that comes from its antibacterial effects was tested in various areas, including the preservation and shelf-life elongation of raw milk, pasteurized milk [11], cheese [16], pork cubes, pork ham, pâté (a kind of meat products) [12], chicken thigh meat [15], and chicken breast fillet [17], and was tested in oral health studies [14,18,19].

#### 3.1.1. Antibacterial Properties of LPO in Milk and Milk Products

Milk takes a massive role in human life due to its nutritional properties, but at the same time, milk is a suitable medium for the growth and proliferation of various microorganisms; this situation is causing its rapid deterioration [90]. Contaminant bacteria can proliferate quickly, making the milk inappropriate for processing and unsuitable for consumption by humans. The use of cooling facilities is the most frequently used preservation method to prevent or slow down the deterioration of milk and minimize postharvest losses from farms to collection centers. Unfortunately, milk collection centers face tough challenges and obstacles such as electrical problems, the lack of available capital, bad road infrastructures, high operating expenses, regular equipment malfunctions, problems in supplying backup parts, and problems with equipment repairs [3]. The potential of LPO in the preservation of and prolonging the shelf life of milk and dairy products attracted attention [11,13,16] due to its antibacterial activity [10,11,12,13,14,15,16,17,18,19,20] and especially because it is already present naturally in milk [6,53,54,55,56,57,58].

In a recent study, a group examined the potential of LPOS to improve the storage life of raw milk and pasteurized milk in regions where there is a lack of cooling systems to store and transfer the milk. Two hundred fifty milk samples, both LPOS-activated morning and LPOS-activated overnight samples, from farmers, collectors, and factories were tested via their total bacterial count (TBC), total coliform count (TCC), and *Escherichia coli* count (EC). The quality of the LPOS-activated milk samples was found to be greater than that of all the control samples, according to the results [11].

In a study, the effects of LPOS that is activated via H_2_O_2_-producing lactic acid bacteria (LAB) on the storage stability of raw camel milk at room temperature were examined and evaluated using acidification curves, titratable acidity (TA), the total bacterial count (TBC), and the coliform count (CC) at 0, 6, 12, 18, 24, and 48 h. *E. coli* growth was used as a contaminating agent in both pasteurized and boiled camel milk samples to obtain data about LPO activity and the inhibitory effect of the LPOS. *Lactococcus lactis* 22333, *Weissella confusa* 22308, *W. confusa* 22282, *W. confusa* 22296, *S. Infatarius* 22279, and *S. lutetiensis* 22319 strains were used in the experiment, and their H_2_O_2_-producing properties were examined. According to the results, *W. confusa* 22282 was found to be the best option to produce H2O2. In some points, exogenous H_2_O_2_ was used at different levels to optimize the LPOS activity. According to results, the LPOS activated by H_2_O_2_-producing LAB have significantly positive effects on the storage of raw camel milk [13].

In a study, LPOS and lysozyme were used to improve the shelf life of dangke, which is a kind of cheese traditional dairy product made of bovine and buffalo milk from Indonesia. The LPOS consists of 300 μL of LPO, 300 μL of 0.9 mM H_2_O_2_, and 300 μL of 0.9 mM KSCN. The quality of the product was evaluated by measuring the pH value, total microbial count, and hardness. The solutions that consist of LPOS, lysozyme, LPOS + lysozyme, and pure water to the control were used to immerse the dangke. The results showed that there is a significant inhibition of the growth of microbes in LPOS-, lysozyme-, and LPOS + lysozyme-immersed dangke samples stored for 8 h; higher antimicrobial activity was observed in LPOS + lysozyme-immersed dangke samples than other samples [16].

In conclusion, studies indicated that the LPOS has great potential to be used for the preservation and prolongation of the shelf life of raw milk, pasteurized milk [13], and milk products such as cheese [16]. In this way, the usage of H_2_O_2_-producing lactic acid bacteria can be an alternative method to activate the LPOS and elongate the storage time of milk without deterioration [13]. All of these studies indicated that the LPOS is promising in the dairy industry, especially in places that suffer from insufficient cooling systems for the storage and transportation of milk and milk products, but more studies are needed to achieve an end product as a commonly used method.

#### 3.1.2. Antibacterial Properties of LPO in Meat and Meat Products

There are various known chemicals used to preserve and elongate the shelf life of meat and meat products, but they are threatening due to risks of negative consequences for human health [12]. At this point, LPO becomes well-known because it is effective against bacteria [2,3,10,11,12,13,14,15,16,17,18,19,20], viruses [3,21,22,23], and fungi [3,25]. It is also found naturally in mammals [6,52,53,54,55,56,57,58].

Another recent study showed that the LPOS can be used to prolong the shelf life of meat and meat products. In this study, the effect of LPO on selected microorganisms was tested in vitro using liquid broth and ex vivo using pork cubes, pork ham, and pâté in the presence of LPO. In the in vitro part of the study, the inhibitory effect of LPO on the growth of *Listeria innocua*, *Staphylococcus saprophyticus*, and *Pseudomonas fluorescens* was tested in liquid broth, and the results showed that LPO exhibits inhibitory activity on these microorganisms due to the prolongation of lag phases. The ex vivo part of the study examined meat (pork cubes) and meat products (pork ham and pâté), as mentioned in Table 3, and in the presence of LPO solution (5 g LPO in 100 mL distilled water) *+ Listeria innocua* for pork cubes and 0.25%, 0.50% LPO for meat products. According to the results of a study on pork cubes, a significant difference was observed in the total viable count (TVC) between LPO-soaked pork cube samples and the control samples; TVC values of LPO-soaked pork cubes were significantly lower than the control samples. According to the results of a study on pork ham and pâté, a significant lowering for values of TVC and lactic acid bacteria (LAB) was observed for both concentrations of LPO, 0.25% and 0.50%, compared to the control samples. Also, a similar positive effect of LPO was observed on the oxidation grade of the products. Totally, the results indicated that the LPO is promising for use in elongating the shelf life of meat and meat products via its antimicrobial activity [12].

In a study, the authors tried to produce an edible coating that consists of whey protein and alginate with the LPOS for improving the shelf life of chicken thigh meat in refrigerated conditions (4 ± 1 °C). The whey protein–alginate coatings were produced without the LPOS and with concentrations of 2%, 4%, 6%, and 8% (*v*/*v*) LPOS. The LPOS that was used in this experiment contained LPO, GO, Glu, KSCN, and H_2_O_2_ in the ratio of 1.00:0.35:108.70:1.09:2.17, respectively. Overall, whey protein–alginate coating incorporated with the LPOS exhibits a significant antibacterial effect, and this study shows that the antibacterial effect increases if the concentration of the LPOS is increased, so the highest antibacterial effect is observed in the coating that includes 8% LPOS [15].

In a study, the effects of alginate coating without LPOS and alginate coating incorporated with LPOS on the storage of chicken breast filets in cold storage were tested. LPOS-containing alginate coatings were produced with 2%, 4%, and 6% (*v*/*v*) LPOS concentrations. The coating was applied to chicken breast filets by immersing them into two solutions. The first solution was made of 5 g alginate, 25 g glycerol, 45 g maltodextrin, and 450 mL distilled water, and the second solution was made of 0.6 g carboxymethyl cellulose, 2.75 g calcium chloride, and 49 mL distilled water. The second solution was combined with the LPOS in different concentrations: 2%, 4%, and 6%. According to the results of the study, the combinations of the LPOS and alginate coating exhibited a positive effect on increasing the shelf life of the chicken breast filets under refrigerated conditions; the coating, which contains 6% *v*/*v* LPOS, exhibited more effectiveness than the samples with 2% and 4% LPOS, especially on the bacteriological side [17].

In conclusion, LPO is a potential alternative to chemicals used for preserving meat and meat products. Studies especially indicated that it can be used as a component of a coating to preserve and elongate the shelf life of chicken meat [15,17]. Additionally, LPO exhibited great antibacterial activity when meat and meat products were soaked in it. Additionally, LPO was successful in inhibiting *Listeria innocua*, *Staphylococcus saprophyticus*, and *Pseudomonas fluorescens* [12]. Despite the positive results of the studies demonstrating LPO’s great potential, further research is necessary to establish LPO as a common tool in the meat industry.

#### 3.1.3. Antibacterial Properties of LPO in Oral Health

LPOS is naturally present in human saliva, and it is one of the main defense systems against dental caries. LPOS regulates the composition of microflora and prevents pathogenic microorganisms found in periodontitis. Due to these properties of LPO, it can be a potential treatment against oral diseases such as dentinal caries and periodontitis, and it can be used in a daily oral hygiene routine. The antibacterial properties of the LPOS especially make it a promising tool against dentinal caries because the bacteria *Streptococcus mutans* is one of the most mentioned reasons for dentinal caries, and LPO can be effective against it too [48].

In a study, the effects of hydroxyapatite–lysozyme–lactoferrin–LPO combination on *Streptococcus mutans* were examined to explore its potential for treatment of dentinal caries. *S. mutans* counts were determined before treatment and 24 h, 1 month, and 6 months after the treatment. In counts, a significant reduction in *S. mutans* was observed at 24 h after treatment in comparison with 1 month and 6 months after. According to the results, it can be said that the combination may be used against *S. mutans* in dentinal caries [14].

In a clinical study, the effect and potential of lozenges that contain LPOS on oral care were examined. Mouth rinse using Listerine as positive control (A) and lozenges that contain 10 mg LPO 350 U/mg, 7.5 mg KSCN and 0.083% H_2_O_2_ in a ratio of 1:2 H_2_O_2_/SCN^−^ (B), 10 mg LPO 350 U/mg, 7.5 mg KSCN and 0.040% H_2_O_2_ in a ratio of 1:4 H_2_O_2_/SCN^−^ (C), and placebo lozenge (D) were used for 4 days instead of the typical daily oral care routine of volunteers. According to the results, the comparison of plaque regrowth values is D > C > B > A, bacterial count values of *Streptococcus mutans* are A > C > D > B, bacterial count values of *Lactobacilli* are A > D > B > C, and the total bacterial count is C > D > B > A. Additionally, an increase in the amount of SCN^−^ values was recorded in B and C groups day by day. The study showed that the LPOS may be used in the daily oral care of humans via its plaque regrowth inhibition and cariogenic bacteria reduction properties [18].

In another clinical study, tablets that contained LF and LPO were tested on the oral health of humans. In this study, subjects were divided into three different groups, and all groups were treated with three tablets per day for 12 weeks; the tablets were used by dissolving them in the mouth. The first group was tested with high-dosage tablets, which contained 60 mg/d LF and 7.8 mg/d LPO; the second group was tested with low-dosage tablets, which contained 20 mg/d LF and 2.6 mg/d LPO; and the third group was tested with placebo tablets. The gingival index (GI), plaque index (PI), and Oral Health Impact Profile (OHIP) were used to show the effectiveness of the treatment. A significant reduction in the GI was observed in the high-dosage group compared to the placebo group after 12 weeks of treatment. A significant reduction in the PI was observed in the high-dosage and the low-dosage groups at 12 weeks compared to the baseline. A significant decrease in the OHIP was observed at 12 weeks in the high-dosage group. The results showed that LF- and LPO-containing tablets may be beneficial to boost the oral health of the humans [19].

In a study from 2025, the effects of LPO, both alone and with XO, on infant oral microbiota were tested in vitro. Investigators carried out the study on bacteria isolated from the oral cavities of five breastfed infants. The results showed that the use of LPO, both alone and incorporated with XO, exhibited significant, dose-dependent antibacterial activity on the tested bacteria. These findings imply that LPO may have antibacterial properties that extend beyond its enzymatic function. An analysis of gene expression revealed that treatment with LPO-XO resulted in the downregulation of several genes associated with ROS, indicating a transient stress response in the bacteria. Additionally, the study noted a downregulation of critical enzymes involved in glycolysis, suggesting that the LPO-XO system also influences bacterial metabolism at the transcriptional level [88].

In conclusion, the studies on the effects and potential of LPO on oral health showed that LPO has a significant antibacterial effect against *Streptococcus mutans* [14,18], and it significantly reduces plaque regrowth [18,19]. Also, LPO can be combined with other active milk proteins, such as lysozyme and LF, to increase the benefits and can be used in a lozenge form to boost oral health, including gingival health, and prevent plaque formation [19,48]. In vitro and in vivo studies showed that LPO reduces adhesion, biofilm viability, and plaque formation and inhibits bacteria (especially *Streptococcus mutans*) [48]. Due to its natural occurrence in saliva [48,52] and antibacterial properties, LPO has great potential to be a part of oral care routines of humans; for example, it can be used as a component of an oral care product such as toothpaste, mouth rinse liquid, or lozenges due to its significant effects. Although the studies have positive indications, more study is needed to use LPO as a health care product.

### 3.2. Antiviral Properties of LPO

Currently researchers have vaccines as a prophylactic option against some viruses, but it brings some challenges such as strain-specific vaccination yearly, the development of resistance, and changes in viruses related to antigenic drift and viral reassortment [21]. There is a lack of alternative options for treatments of viral diseases. At this point, LPO gains importance as an alternative approach for the treatment of viral infections. LPO treatment may be cheaper than current treatments, and it may not have to deal with the challenges that vaccines and other treatments have to face.

In a study, the sensitivity of the 12 different influenza virus strains to OSCN^−^ and hypoiodite (OI^−^) was examined in vitro using a system that contains three components: the LPOS, glucose, and GO. The results showed that all strains are inactivated by OSCN^−^ and OI^−^ but at different inactivation levels and with different substrate preferences. While OSCN^−^ provided more inactivation to some strains than OI^−^, some strains were more affected by IO^−^ than OSCN^−^, and some strains had the same sensitivities to both LPO substrates. In the study, OSCN^−^/OI^−^ susceptibility ratios of the influenza A strains, and influenza B strains were examined, and the results showed that the ratio is much higher in influenza A viruses than in influenza B viruses. Eventually, the LPOS may be used against influenza A strains and influenza B strains [21].

In a study, the inhibitory effects of the LPO, LF, angiogenin-1, α-lactalbumin, β-lactoglobulin, casein, lactogenin, and glycolactin on human immunodeficiency virus-1 reverse transcriptase (HIV-1RT), α-glucosidase, β-glucosidase, and β-glucuronidase were tested; also, the effects of the succinylation of these proteins on their inhibitory effect were tested. The comparison of inhibitory effects of the proteins on HIV-1RT was bovine LF > human LF > LPO > lactogenin > angiogenin-1 > glycolactin > β-lactoglobulin, while α-lactalbumin and casein did not exhibit any inhibitory actions on HIV-1RT. According to the results, succinylation improved the inhibitory effects of human lactoferrin, glycolactin, and β-lactoglobulin, and it gave inhibitory effects to α-lactalbumin and casein. Eventually, this study indicated that LPO and some other milk proteins may be used for the treatment and prevention of HIV-1. Additionally, the concentrations of the proteins that reached 50% inhibition of HIV-1RT were given as 6.5 μM for lactoferrin, <55 μM for LPO, <78 μM for glycolactin, <290 μM for lactogenin, and <330 μM for angiogenin-1 in the study [22].

A study indicated that high dietary iodine intake may be beneficial against COVID-19. In this study, a compilation that includes the population, vaccination ratio, infection ratio, and rate of deaths caused by COVID-19 of some countries was prepared. The compilation showed that in Asian countries that have high-iodine-containing eating habits, such as Japan, South Korea, and India, death rates caused by COVID-19 were significantly lower than that in western countries like the United States, the United Kingdom, and Sweden. This situation sparked an idea that the difference may come from the difference between the iodine intake of the countries, and the high dietary iodine intake may contribute to protection from viruses via the relation between iodine and LPO [23].

In a study, antiviral activities of bovine milk LPO, camel milk LPO, and human colostrum LPO against Herpes Simplex Virus Type 1 (HSV-1) were examined in vitro. In this study, a monolayer of vero cells was infected with 25 PFU HSV-1 pretreated with either bovine milk LPO, camel milk LPO, or human colostrum LPO at concentrations of 0.1, 0.2, 0.3, 0.4, or 0.5 mg/mL in the presence of 5 µL H_2_O_2_ and 10 mg NaSCN, then incubated at 37 °C for 3 days. The results showed that the activity of bovine milk LPO against HSV-1 was 24, 38, 62, 80, and 100%; that of human colostrum LPO was 10, 16, 30, 44, and 66%; and that of camel milk LPO was 12, 18, 34, 50, and 70% at 0.1, 0.2, 0.3, 0.4, and 0.5 mg/mL concentrations, respectively [85].

In a different study, antiviral protection and neutralization effects of bovine LPO, camel LPO, and human LPO against hepatitis C virus genotype 4 were tested. To examine the protective effect, purified bovine, camel, or human LPO was added to HepG2 cells to a final concentration of 0.5 and 1.0 mg/mL, then incubated; after that, free bovine, camel, and human LPO were removed, and a medium containing HCV-infected serum was added and then cultured for 7 days. To examine the neutralization effect, bovine, camel, or human LPO were incubated with HCV-infected serum at concentrations of 0.5, 1.0, and 1.5 mg/mL; then, the mixture was added to HepG2 cell culture, and the inoculated cells were incubated for 7 days. To examine the effects of bLPO, cLPO, and hLPO on HCV replication in the HCV-infected HepG2 cells, HepG2 cells were infected with HCV, and then, purified bLPO, cLPO, or hLPO samples were added to the final concentrations of 0.25, 0.50, 0.75, 1.0, 1.25, and 1.5 mg/mL. According to the results, all proteins were insufficient in protecting the HepG2 cells from the HCV entry at tested concentrations. In the neutralization part, bovine and human LPO completely neutralized the HCV particles and inhibited the HCV entry at a concentration of 1.5 mg/mL, and camel LPO showed the same effect at concentrations of 1.0 and 1.5 mg/mL. In the neutralization part, bovine and human LPO failed at concentrations of 0.5 and 1.0 mg/mL, while camel LPO failed at a concentration of 0.5 mg/mL [84].

To sum up, studies indicated that LPO-containing treatments showed significant antiviral activity against many viral strains, such as 12 different influenza strains [21], human immunodeficiency virus-1 [22], Herpes Simplex Virus Type 1 [85], hepatitis C virus genotype 4 [84], and potentially against COVID-19 [23]. Although products of LPO-catalyzed reactions, OSCN^−^ and OI^−^, showed inhibitory effects at different levels on some influenza strains, both were significantly effective, and some strains were equally sensitive to them [21]. Additionally, studies indicated that there are differences in the effects of different animals’ LPO enzymes on tested strains [84,85]. Bovine milk LPO was more effective than camel milk LPO and human colostrum LPO on Herpes Simplex Virus Type 1; bovine milk LPO at a 0.5 mg/mL concentration completely inhibited the viral activity, while at the same concentration, camel milk LPO and human colostrum LPO could not inhibit it entirely. At lower concentrations, bovine LPO was still more effective than at the same concentration of camel milk LPO and human colostrum LPO [85]. In neutralizing the hepatitis C virus and inhibiting its entry, camel LPO was more successful than bovine LPO and human LPO. At 1.5 mg/mL concentration, human LPO and bovine LPO completely neutralized the HCV particles and inhibited HCV entry. Camel LPO at 1.0 mg/mL concentration showed the same effect, while human LPO and bovine LPO did not [84]. LF and LPO both had a potent inhibitory effect on human immunodeficiency virus-1 reverse transcriptase [22]. This means that the two chemicals could be used together to treat HIV infection. In conclusion, LPO is a great applicant for the treatment of viral infections, but it should be studied strain-specifically.

### 3.3. Antifungal Properties of LPO

Postharvest crop losses, including fruits and vegetables, are a common problem that causes a loss of money and sources. One of the main reasons for this situation is fungal activity. To counteract this problem, humans developed some strategies, such as fungicidal chemicals [80], but it brought some challenges, such as fungicide resistance [91,92,93]. Fungicides are a class of pesticides. The use of fungicides has known harmful effects, such as human toxicity and ecotoxicity [94]. At this point, LPO may be a beneficial alternative due to its wide antimicrobial spectrum [2,3,10,11,12,13,14,15,16,17,18,19,20,21,22,23,24,25,81,82,83,84,85].

In a study, candidacidal activities of the GO mediated LPOS on *Candida albicans* ATCC strains 18804, 10231, and 11006 were tested by using bovine LPO (25 and 50 µg/mL), KSCN (1 mM), GO (1, 5, 10, and 20 units/mL), and glucose (0.03, 0.3, and 3.0 mg/mL). The candidacidal activity of the system was tested without preincubation and by preincubating the system components for 30 or 60 min. After preincubation, Candida albicans cell suspension was added; the mixture was incubated, then diluted, and the diluted cells were plated onto the plates. Candidacidal activity levels were varying between the strains and the activities of the systems were increasing when the preincubation times were increased. When the system consisted of 25 µg/mL of bovine LPO, 10 unit/mL of GO, and 0.03 mg/mL of glucose was tested, results showed that the loss of viability without preincubation was 13.9–27.4% and with incubation for 60 min was 28.6–34.3%. The highest candidacidal activity by the system was observed on *Candida albicans* ATCC strain 11006. Additionally, the usage of only bovine LPO and KSCN (in the absence of GO and glucose) or of bovine LPO, KSCN, and glucose (in the absence of GO) showed candidacidal activities that were lower than those of complete systems. When 25 µg/mL of bovine LPO, 1 mM of KSCN and 10 units/mL of GO was tested at different glucose levels (0.03, 0.06, 0.3, and 3.0 mg/mL) without preincubation and with preincubation for 30 or 60 min; the results showed that candidacidal activity of the system at 3.0 mg/mL glucose was much more than that of lower glucose concentrations. At the 3.0 mg/mL glucose level, the candidacidal activity reached about 100% when preincubation was applied for 30 or 60 min. In conclusion, the results demonstrated that the candidacidal activity of the system depends on the strain and preincubation [24].

In a study, the influence of chitosan coating with or without the LPOS on postharvest mangoes was tested. Iodine containing the LPOS and the LPOS without iodine were tested in the experiment. Chitosan at concentrations of 0.5, 1.0, and 1.5% was tested. The LPOS was prepared with LPO, GO, Glu, KSCN, and KI in weight ratios of 0.35, 1.00, 1.09, 2.17, and 108.70, respectively. Strains of *Colletotrichum gloeosporioides*, *Phomopsis* sp. RP257, *Pestalotiopsis* sp., and *Lasiodiplodia theobromae* ngr 05 A were used in the study. When the LPOS or LPOSI incorporated with chitosan, the inhibitory effects increased. A significant difference between the effects of the LPOS and LPOSI at the same concentration of chitosan was not observed, so the iodine content in the LPOS could not significantly affect the antifungal performance of the LPOS. Inhibition by the coatings was increased by the LPOS in all cases. Moreover, some chitosan-resistant strains became more susceptible to the LPOS. For example, when 1% chitosan alone was used on *Phomopsis*, the inhibition was 58% while that was 100% when the LPOS is present. The findings suggested the presence of a synergistic effect of the LPOS and chitosan [82].

In a study, antifungal activity of the LPOS consisted of goat milk LPO, SCN^−^, and H_2_O_2_ was tested on strains of *Aspergillus niger*, *Pencillium chrysogeum*, *Aspergillus flavus*, *Alternaria* sp., *Trichoderma* sp., *Corynespora cassiicola*, *Phytopthora meadii*, *Claviceps* sp., *Corticium salmonicolor*, *Candida albicans*, and *Pythium* sp. *Candida albicans* and *Pythium* sp. were resistant to the goat milk LPO-SCN^−^-H_2_O_2_ system. MIC values of goat LPO in the system against the strains tested were 475 µg/mL for *Aspergillus niger*, 490 µg/mL for *Pencillium chrysogeum*, 484 µg/mL for *Aspergillus flavus*, 493 µg/mL for *Alternaria* sp., 244 µg/mL for *Trichoderma* sp., 483 µg/mL for *Corynespora cassiicola*, 119 µg/mL for *Phytopthora meadii*, 62 µg/mL for *Claviceps* sp., and 242 µg/mL for *Corticium salmonicolor* [81].

In a study from 2020, the antifungal activity of the LPOS against two strains of fungi, *Phomopsis* sp. RP257 and *Pestalotiopsis* sp., isolated from mangoes, was tested using an edible coating, which is made of chitosan and the LPOS. The coating solution was tested in vitro and in vivo. The LPOS solution used in coating was prepared with 15.5 mg of LPO, 10.6 mg of GO, 3.36 g of glucose, 32 g of potassium thiocyanate, and 55 mg of potassium iodide in 50 mL of buffer phosphate at pH 6.2 (0.1 M). Two LPOS solutions were prepared in the study: one was prepared without iodide, and the other one was prepared with iodide. Different chitosan solutions were prepared by dissolving the chitosan in distilled water containing 0.7% lactic acid (*v*/*v*) at pH 5.5 by setting 0.46M K_2_HPO_4_ in different concentrations of 0.5, 1.0, and 1.5% (*w*/*v*). Then, glycerol (25% *w*/*w* of chitosan) was added into the solution. After that, the LPOS solutions were incorporated with the chitosan solution in a way that the LPOS solution concentration of the mixture was 5% (*v*/*v*). In the end, three solutions were obtained for each chitosan concentration: chitosan solution containing LPOS solution without iodide, chitosan solution containing LPO solution with iodide, and chitosan solution without LPOS solution. In the in vitro part of the study, the disk diffusion method was used to test the antifungal activity. According to the results of the in vitro part, 0.5% chitosan without the LPOS exhibited a low inhibitory effect against *Pestalotiopsis* sp., while the 1.0% and 1.5% ones inhibited the growth totally; however, when the LPOS with or without iodide was used with 0.5% chitosan, the inhibition reached 93% from 26%, while the LPOS with or without iodide did not significantly influence the inhibition level of 1.0% and 1.5% chitosan. On the other hand, *Phomopsis* sp. RP257 was resistant to chitosan without the LPOS, but the resistance was decreasing when the chitosan concentration was increasing. Also, the incorporation of chitosan with the LPOS without iodide increased the inhibition, while chitosan with the LPOS with iodide exhibited a low inhibition level. In the in vivo part of the study, in both strains, an increment was observed in the inhibition level when the chitosan concentration increased or the LPOS with or without iodide was added. The *Phomopsis* RP257 strain was more resistant to coating than the *Pestalotiopsis* sp. strain. *Pestalotiopsis* sp. was inhibited at 100% in chitosan concentrations of 1.0 and 1.5%, while *Phomopsis* RP257 was inhibited at about 50% at the same concentrations. Additionally, chitosan solutions that contained the LPOS without iodide exhibited higher inhibition than chitosan solutions that contained the LPOS with iodide against *Phomopsis* RP257. In conclusion, this study showed that the LPOS incorporated with chitosan can be used to prevent postharvest issues such as stem and rot caused by fungal microorganisms, specifically *Pestalotiopsis* sp. and *Phomopsis* RP257, especially the LPOS without iodide [25].

In a study, synergistic anti-candida activities of LF and the LPOS on *Candida albicans* were tested by using bovine LF and bovine LPO. The LPOS was prepared with 43 mg/g bovine LPO, 430 mg/g GO, 450 mg/g glucose, 24 mg/g citric acid, and 53 mg/g sodium citrate. The LPOS and LF both affected the growth morphology of *Candida albicans.* The mycelial volume of *Candida albicans* was not reduced by bovine LF alone at 500 µg/mL, while morphologically, the hyphal length of *Candida albicans* was shorter than those of the control. On the other hand, the LPOS alone at the same concentration morphologically changed the hyphal shape of *Candida albicans* to a more isolated and smaller colony-like appearance. The combination of 125 µg/mL of bovine LF and 125 µg/mL LPOS changed the size and shape of cells and notably reduced the mycelial volume. The cellular metabolic activity of *Candida albicans* was not affected by the LPOS and LF when they were used alone at a concentration of 125 µg/mL. LF alone did not exhibit an inhibitory effect, even at 2000 µg/mg. Additionally, the LPOS alone at 500 µg/mL showed a weak inhibitory effect that was 30.7%. Other than that, the metabolic activity of *Candida albicans* was completely stopped when LF at concentrations higher than 7.8 µg/mL and the LPOSs at concentrations higher than 31 µg/mL were mixed together. LF and the LPOS both significantly affected the adhesive hyphal form of *Candida albicans.* The IC_50_ of LF alone was 1000 µg/mL, while that of LPOS was 400 µg/mL. On the other hand, the combination of LF at 7.8 µg/mL and the LPOS at 50 µg/mL showed 82.6% inhibition. The effect of the combination of 1000 µg/mL LF and 600 µg/mL LPOS was 90.8%. In conclusion, LF and the LPOS can significantly influence *Candida albicans*, and they have a significant synergistic relationship against fungi [83].

To sum up, studies indicated that LPO has great antifungal activity on many strains such as *Phomopsis* sp. RP257, *Pestalotiopsis* sp. [25,82], *Colletotrichum gloeosporioides*, *Lasiodiplodia theobromae* ngr 05A [82], *Aspergillus niger*, *Pencillium chrysogeum*, *Aspergillus flavus*, *Alternaria* sp., *Trichoderma* sp., *Corynespora cassiicola*, *Phytopthora meadii*, *Claviceps* sp., *Corticium salmonicolor*, *Candida albicans* and *Pythium* sp. [81], and *Candida albicans* ATCC strains 18804, 10231, 11006 [24]. Researchers prepared a coating using LPO and chitosan, a substance known for its antimicrobial properties, including its ability to combat fungi. According to the results, often, LPO increased the inhibitory effect of the coating on the fungal strains tested [25,82]. In conclusion, the use of LPO against fungi that cause plant diseases, particularly to maintain crop yield, holds great potential. Additionally, combining chitosan and LPO can enhance its effectiveness against various fungal strains.

### 3.4. Other Properties of LPO

Various functions of LPO were reported and identified [2,3,10,11,12,13,14,15,16,17,18,19,20,21,22,23,24,25,81,82,83,84,85] thanks to the fact that LPO has been investigated for decades [2,11,12,50,95,96]. In addition to its well-known antibacterial, antiviral, and antifungal properties, new properties and functions of LPO and new strains that are sensitive to LPO are still being discovered.

In a study from 2024, researchers used adult male rats to examine the antioxidant and therapeutic effects of LPO on nephrotoxicity caused by aflatoxin B1 (AFB1). This study involved 40 adult male rats divided into four groups in a way that each involved 10 rats. The first group was the control group; the second group was treated intraperitoneally with LPO 50 mg/kg/day for 6 weeks; the third group was intoxicated orally by giving them AFB1 80 µg/kg/day for 6 weeks; and the fourth group was treated intraperitoneally with LPO 50 mg/kg/day for 6 weeks after being intoxicated with AFB1 80 µg/kg/day for 6 weeks. The researchers measured the markers of kidney function such as creatinine, blood urea, urea nitrogen, sodium, potassium, magnesium, total calcium, phosphorus, and albumin. Measurements showed that there are significant differences between groups 1 and 3 and between groups 3 and 4. According to the results, the values of group 4 were significantly closer to the values of group 1 compared to group 3. This situation indicated that LPO treatment after AFB1 intoxication in group 4 approached all the values of group 1, which consisted of healthy individuals. Researchers also measured kidney values such as malondialdehyde (MDA), nitric oxide (NO), reduced glutathione (GSH), superoxide dismutase (SOD), and catalase (CAT). The same situation was observed in this part as well. There were significant differences between groups 1 and 3 and between groups 3 and 4; furthermore, the results indicated that LPO treatment after AFB1 intoxication ameliorated the deteriorations, so it approached all values in group 1. Other parameters that were examined in the study were immune system-related components such as TNFα, IL1β, CD4, and DNA fragmentation. When these parameters were examined, the same results were observed as well. Significant differences were observed between the groups 1 and 3 and between the groups 3 and 4 for these four parameters; furthermore, LPO treatment ameliorated the deteriorations caused by AFB1 intoxication as well as in the previously mentioned results of other measurements. In conclusion, the study showed that LPO has a therapeutic potential for the treatment of nephrotoxicity and tissue degeneration caused by AFB1 [87].

In a study from 2021, researchers deleted the LPO gene from mice, and they obtained interesting results that help to understand LPO and its importance. The researchers observed some diseases in LPO gene-deleted mice at significantly higher frequencies than in wild-type mice. The results showed that the deletion of the LPO gene caused significantly higher rates of cardiomyopathy, carditis, arteriosclerosis, airway inflammation, glomerulonephritis, and inflammation in digestive system organs such as the small intestine, colon, liver, and pancreas. Researchers also reported that they observed significant brain pathology, including ventriculomegaly, degenerative changes, and neuroaxonal dystrophy. Another striking result was that tumor presence was detected in 7 mice from a group of mice consisting of 19 one-year-old LPO gene-deleted mice; the tumors were various, including carcinoma within the lung; lymphoma adjacent or attached to the heart, mesentery, pancreas, salivary glands, or lung; in the spleen or small intestines; pleomorphic sarcoma in the skin; and histiocytic sarcoma in the spleen, liver, or bone marrow, while none of them were detected in the wild-type mice used in the study. Additionally, some LPO gene-deleted mice were overweight or even obese. This study showed the importance of LPO genes and that there are still some facts about LPO waiting to be revealed [86].

In a study from 2025, bovine milk LPO incorporated with a nanocomposite (GRO-PAA-Cu) consisting of graphene oxide (GRO), polyacrylic acid (PAA), and Cu was tested to investigate LPO’s effects on the anticancer properties of the composite. Initially, GRO was radically anchored with PAA, followed by activation through the substitution of the H- atom in the carboxyl group of PAA with a sodium ion. Subsequently, copper ions were efficiently loaded onto the activated carboxylated groups, where they function as a catalyst in conjunction with LPO. The resulting composites were extensively characterized using a variety of analytical techniques, including SEM, TEM, EDX, FTIR, TGA, and XRD. Additionally, the zeta size and potentials for each composite were determined. The experimental findings demonstrated that LPO in the modified form (GRO-PAA-Cu-LPO) maintained its stability under storage conditions, retaining approximately 73% of its original activity after 9 weeks of storage at 4 °C. Furthermore, the data revealed that the GRO-PAA-Cu-LPO composite exhibited greater selectivity towards both Caco-2 and Huh-7 cells compared to the free GRO-PAA-Cu composite and free LPO. These results suggest that the incorporation of LPO into the modified GRO-POO-Cu composite significantly enhanced its selectivity against all treated cancer cells. Additionally, the modified GRO-PAA-Cu-LPO complex was found to promote cell cycle arrest in treated cancer cells, particularly in the sub-G1 (apoptotic) and S phases, when compared to untreated cells, thereby facilitating the apoptotic mechanism [89].

A study from 2022 showed that LPO activity may be measured to determine the mammary gland health of cows due to LPO activity increases when mastitis occurs in cows [31]. These results indicated that LPO has a great potential in other various areas due to its other properties in addition to its antibacterial, antifungal, and antiviral activity. LPO may be a common tool, but more studies are needed to clarify its potential.

## 4. Potential Applications of Lactoperoxidase

LPO has a great antibacterial effect on many bacterial strains, and it can be an alternative treatment against diseases caused by bacteria [10,11,12,13,14,15,16,17,18,19,20]. It can be used instead of antibiotics [10], which have many known side effects, including diarrhea, *Clostridium difficile* infection (CDI), the selection of antibiotic-resistant microorganisms, allergy, and obesity [97]. LPO-catalyzed reactions can be used even against drug-resistant bacteria [20]. However, oral usage of LPO instead of antibiotics or adjunct to antibiotics brings some challenges, such as degradation by digestive system. LPO may be used in topical applications, such as wound care, eye drops, and skin infections, since these forms do not come into contact with the digestive system. Also, it may be applied to the surfaces with infection risks, such as catheters or wound dressings, to prevent bacterial contamination. Furthermore, some methods, such as appropriate protective formulation or recombinant DNA technology, to produce more resistant forms of LPO may be developed and used to avoid inactivation in the gastrointestinal tract.

**Figure 3:**
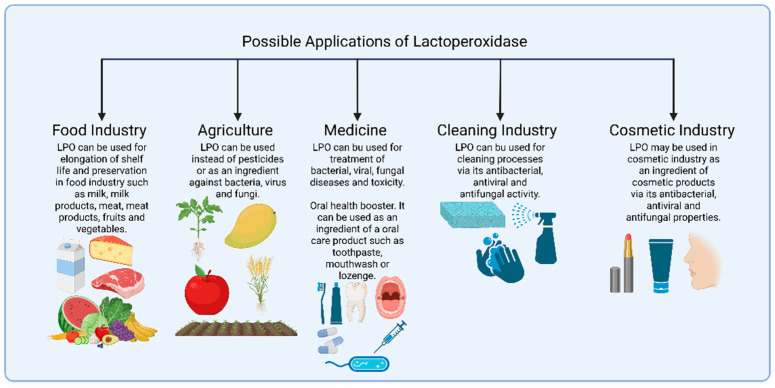
A demonstration shows possible applications of LPO.

One of the most suitable areas of use for LPO is the dairy industry. LPO is already found in milk; thus, it is a safer option for maintaining the quality of milk and milk products [6,53,54,55,56,57,58]. Currently, one of the most commonly used techniques for the preservation and shelf-life elongation of milk in the dairy industry is heat treatment, which causes a significant reduction in the nutritional values of milk, especially proteins and water-soluble vitamins [98]. LPO could be a good alternative at this point, or it could let pasteurization processes happen at lower temperatures, which would mean that milk loses fewer of its nutritional properties during pasteurization. There would be no difference in the amount of microbes present in milk that was pasteurized at higher temperatures compared to milk that was pasteurized at lower temperatures. For this purpose, LPO/H_2_O_2_/SCN^−^ and LPO/H_2_O_2_/I^−^ systems, which have known antimicrobial activity, can be used; their components can be added into the milk to boost the benefits of the LPO (Figure 3).

Additionally, when a mother’s milk is not present or not enough, the best option is to acquire donor milk. The milk obtained from human milk banks is usually pasteurized using Holder pasteurization (performed at 62.5 °C for 30 min), which is a technique recommended in international guidelines. Although it is performed at lower temperatures than those of other common heat-based pasteurization procedures, it causes a significant reduction in the nutritional potential of the milk. Studies indicated that it causes a significant reduction for vitamins such as ascorbic acid + dehydroascorbic acid, ascorbic acid, and vitamin B6; hormones such as insulin and adiponectin; growth factors such as IGF-1, IGF-2, IGFBP-2, IGFBP-3, EPO, HB-EGF, and HGF; enzymes such as lipase, alkaline phosphatase, and amylase; cytokines such as MIP-1β and MCAF/MCP-1; and immunoglobulin G4 [99]. LPO can be a great alternative to provide better nutrition to newborn infants when a mother’s milk is not available or is not enough.

Another potential usage area for LPO is the meat industry. Due to its great antimicrobial spectrum, LPO is a suitable alternative to chemicals used in the meat industry to preserve and elongate the shelf life of meat and meat products. It can be used as an edible coating material, or a method may be developed that allows for the preservation of meat and meat products by soaking them in an LPO-containing solution.

Due to its natural occurrence in the human body, including saliva [48,52], and its antimicrobial properties [2,3,10,11,12,13,14,15,16,17,18,19,20,21,22,23,24,25,81,82,83,85], especially antibacterial [2,3,10,11,12,13,14,15,16,17,18,19,20,24], LPO has a great potential to be used to support oral health and treat many oral diseases, including gingival diseases and dental diseases, or to prevent carious lesion formation in teeth. It may be used in oral hygiene products such as toothpaste and mouth rinse liquids and can be used in a lozenge form. Additionally, it may be combined with other beneficial proteins such as LF to boost antimicrobial effect. LPO has known antiviral activity against many virus strains, such as 12 different influenza strains [21], human immunodeficiency virus-1 [22], Herpes Simplex Virus Type 1 [85], hepatitis C virus genotype 4 [84], and potentially against COVID-19 [23]. According to these data, LPO is a potential enzyme to be used against various viruses; it can inhibit and neutralize them, and thus, many viral diseases may be prevented or treated.

Currently, to maintain crop yield and reduce postharvest losses, chemicals (especially fungicides) are commonly used to prevent or treat diseases in plants, including fruits and vegetables [100]. Fungicides are a class of pesticides, and the usage of fungicides contributes to human toxicity and ecotoxicity [94]. Fungicides can have adverse health effects, particularly when misused. They can cause allergic dermatitis and skin or mucous membrane irritation. Pesticides may potentially cause acute and chronic toxic reactions, mutagenicity, carcinogenicity, and reproductive dysfunction [101]. On the other hand, pesticides, especially fungicides, have negative impacts on agriculturally important insect pollinators. Fungicides cause lethal and sublethal outcomes for pollinators exposed to fungicides [102]. Due to its antimicrobial properties, especially antifungal activity, LPO can be an alternative to pesticides in agriculture.

## 5. Conclusions and Future Remarks

LPO is an important enzyme that is promising in various fields, including the food industry (milk, dairy products, meat, fruits, and vegetables), agriculture, and medicine. In medicine, LPO can be used in oral health as an oral care product or as a treatment product against bacterial, viral, fungal infections, and toxicity. Furthermore, it has potential uses in industrial cleaning and disinfectant products, veterinary purposes (including diagnosis), and many other areas owing to its antibacterial, antiviral, antifungal, antioxidant, anti-inflammatory, anti-plaque, and therapeutic properties. The enzyme’s diverse applications continue to expand with ongoing research.

However, there are some limitations and points that may be improved. New biotechnological methods for more efficient production and purification of LPO may be developed for the future. Further, the effects of different pasteurization techniques on the LPO content of milk should be studied in more detail to determine the best technique. Furthermore, interactions of LPO with other biological molecules should be investigated, especially for use in biological areas. Studies on LPO highlight the need for research on its stability in different environments and its effects on various microorganisms. Further investigations into the enzyme’s biological interactions may enable the development of new treatment methods and biotechnological applications. Moreover, genetic engineering applications and biotechnological innovations may be used to improve the enzyme’s production efficacy, enzymatic activity, and spectrum. Also, further genetic studies and biotechnological developments may make LPO more suitable for broader industrial use. In the future, LPO may be applied in a wider range of fields, as its potential becomes more evident. Additionally, there are some other important points that may be improved such as commercial production of LPO and cost-effectiveness.

In conclusion, LPO has a continually growing and evolving potential in scientific and industrial research. As a result, it is expected that this enzyme will find even more innovative and efficient applications in the future.

## Figures and Tables

**Table 1:** LPO activity in different milks.

	LPO Activity (Units/mL)	Reference
Cow	1.4	[54]
Ewe	0.14–2.38	[55]
Goat	1.55	[56]
	4.45	[57]
Buffalo	0.9	[58]
Guinea Pig	22	[54]
Human	0.06–0.97	[53]
Limit for bactericidal activity	0.02	[2]

**Table 2:** Thiocyanate concentrations of different milks and human extracellular fluids.

	Thiocyanate Concentration	Reference
Cow milk	3.2–4.6 ppm	[67]
Ewe milk	10.3–20.6 ppm	[55]
Goat milk	4.03 ppm	[56]
	10.29 ppm	[57]
Buffalo milk	5.4 ppm	[58]
Human milk	2.6 ppm	[69]
	0.1–4 µM	[65,70]
Human saliva	500–3000 µM	[71,72,73]
Human nasal airway fluid	100–1200 µM	[74]
Human lung airway fluid	30–650 µM	[75,76]
Human gastric fluid	250–300 µM	[77]
Human tears	150 µM	[73]
Human plasma	5–50 µM	[74,78]
Limit for bactericidal activity	15 ppm	[67]

**Table 3:** A comparison of studies that show functions of LPO.

Function	Study Design	Treatment	Results	Reference
Antibacterial	In vitro	Susceptibilities of clinical isolates (*n* = 12) and type strains (*n* = 5) of *Actinobacillus actinomycetemcomitans* to LPO-I^−^-H_2_O_2_ combination and different antibiotics were tested. In combination, 75 µg LPO, 100 nmol I^−^, and 1000 nmol H_2_O_2_ were used.	The combination exhibited an inhibition on *Actinobacillus actinomycetemcomitans* similar to 2 microgram ampicillins.	[10]
Antibacterial	In vitro	Milk samples (*n* = 250) consisting of morning and overnight samples were tested. LPO in raw milk was activated by addition of 14 mL of freshly made NaSCN (1 mg/mL) solution per liter of milk, and in total, 10 mL of freshly made 1 mg/mL H_2_O_2_ solution.	The quality of the all LPOS-activated milk samples was found to be higher than all the control samples.	[11]
Antibacterial	In vitro and ex vivo	In vitro: 14 mL TSB broth, 1 mL inoculum (*Listeria innocua*, *Pseudomonas fluorescens*, or *Staphylococcus saprophyticus*), 1 mL 1% LPO (sterile distilled water). Ex vivo meat: 3 groups of pork (shoulder) cubes (2 × 2 × 2 cm) were soaked in distilled water, TSB broth with *Listeria innocua*, and LPO solution (5 g LPO in 100 mL distilled water). 0.25% and 0.50% LPO solutions were used to make pork ham and pâté ex vivo.	LPO has significant inhibitory effects on growth of *Listeria innocua*, *Pseudomonas fluorescens*, and *Staphylococcus saprophyticus*. Study showed that LPO can be used to elongate shelf life of meat and meat products.	[12]
Antibacterial	In vitro	Specific dosage of LPOS components was not mentioned. Raw camel milk, pasteurized raw camel milk, and boiled camel milk each were tested by itself, with addition of exogenous H_2_O_2_ or with addition of H_2_O_2_-producing lactic acid bacteria.	LPOS activated by H_2_O_2_-producing lactic acid bacteria have significantly positive effects on storage of raw camel milk. *Weissella confusa* 22282 was determined as the best strain of the strains tested in the study to produce H_2_O_2_.	[13]
Antibacterial	In vitro	Challenge 20 permanent third molars to replicate caries-affected dentin. Before sealing, five samples were treated with LPO, LF, lysozyme, and hydroxyapatite. The combination contains 0.018 mg LPO, LF, lysozyme, and hydroxyapatite powders. The total viable Streptococcus mutans count was measured before and 24 h, 1 month, and 6 months after therapy.	LPO may be combined with LF, lysozyme, and hydroxyapatite for treatment of dentinal caries. A significant reduction in *Streptococcus mutans* was observed 24 h after treatment by the combination.	[14]
Antibacterial	In vitro	A whey protein-alginate coating with LPOS was created and tested for its antibacterial properties on chicken thigh meat. The coatings were produced with varying concentrations of LPOS, including LPO, GO, Glu, KSCN, and H_2_O_2_.	A whey protein-alginate coating with LPOS has a substantial antibacterial impact, and this study found that the effect increases with LPOS content, with the largest effect at 8% LPOS. A study found that LPOS coating can extend chicken thigh meat shelf life.	[15]
Antibacterial	In vitro	Dangke samples were immersed in solutions of LPOS, lysozyme, or LPOS + lysozyme at 30 °C for 4 h. Dangke which was immersed in distilled water was used as a control. LPOS was prepared with 300 μL of LPO, 300 μL of 0.9 mM H_2_O_2_, and 300 μL of 0.9 mM KSCN.	The results showed that there is a significant inhibition of the growth of microbes in LPOS, lysozyme, and LPOS + lysozyme-immersed dangke samples stored for 8 h, higher antimicrobial activity was observed in LPOS + lysozyme-immersed dangke samples than other samples	[16]
Antibacterial	In vitro	Effects of alginate coatings with and without LPOS on shelf life of chicken breast fillet were tested. LPOS-containing alginate coatings were produced with 2%, 4%, and 6% (*v*/*v*) LPOS concentrations. LPOS was made of LPO, GO, Glu, KSCN, and KI at ratios of 1.00:0.35:108,70:1.09:2.17 (*w*/*w*), respectively. 15.5 mg LPO was used.	The combinations of LPOS and alginate coating exhibited a significant positive effect on increasing the shelf life of the chicken breast fillets under refrigerated conditions. Higher effect was observed at higher LPOS concentrations.	[17]
Antibacterial	In vitro	The antibacterial activity of a system containing goat LPO, H_2_O_2_, and KSCN was tested on various bacteria by using the disc diffusion method	The system was effective on all tested strains. Inhibition zones were ranging from 22 mm to 26 mm and MIC values for goat milk LPO in the system were ranging from 49 µg/mL to 297 µg/mL	[81]
Antibacterial	In vitro	Effects of bovine milk LPO, both alone and combined with bovine milk XO, on infant oral microbiota were tested. The study was carried out with bacterial cultures from the oral cavities of 5 infants.	Both LPO alone and with XO showed a significant antibacterial effect. According to gene expression analysis, LPO-XO treatment downregulated various bacterial genes associated with ROS. Additionally, downregulation of enzymes involved in glycolysis was observed.	[88]
Antibacterial and Antiplaque	Four-replicate crossover study design	A study involving 16 volunteers was conducted to test four different oral hygiene treatments. The treatments were applied in five different sequences. The control group received a mouth rinse with Listerine twice daily. The B treatment involved lozenges containing 10 mg LPO 350 U/mg, 7.5 mg KSCN, and 0.083% H_2_O_2_ in a 1:2 H_2_O_2_/SCN^−^ ratio, while the C treatment used lozenges with 10 mg LPO 350 U/mg, 7.5 mg KSCN, and 0.040% H_2_O_2_ in a 1:2 H_2_O_2_/SCN^−^ ratio	The study found that LPOS-containing lozenges can be used in daily oral care for humans due to their anti-plaque regrowth and cariogenic bacteria reduction properties. Treatment B reduced more Streptococcus mutans than treatment C and D, while treatment A showed the most significant antiplaque-regrowth activity. Treatment C showed more reduction on Lactobacilli.	[18]
Antibacterial, antiplaque and anti-inflammation	A randomized, double-blind, placebo-controlled clinical trial study design	150 adults were divided into 3 groups and treated by tablets in addition to their daily oral hygiene routine for 12 weeks. The first group was treated with high-dosage tablets that contained 60 mg/d bovine LF and 7.8 mg/d bovine LPO, the second group was treated with low-dosage tablets that contained 20 mg/d bovine LF and 2.6 mg/d bovine LPO, and the third group was treated with placebo tablets.	After 12 weeks of treatment, the high dosage group had considerably lower gingival index (GI) than the placebo group. Both high- and low-dosage groups had considerably lower plaque index (PlI) at 12 weeks than baseline. The high-dosage group had a considerably lower 12-week OHIP score. Results demonstrated that bovine LPO and bovine LF pills may improve oral health in healthy persons.	[19]
Antibacterial and immunomodulatory	In vitro and in vivo	Camel colostrum LPO and camel colostrum LF were tested against 14 multidrug-resistant Acinetobacter baumannii isolates from patients with nosocomial pneumonia. The study used the agar well diffusion method to examine their inhibitory effect. In vivo, 5–6-week-old mice were divided into five groups and exposed to experimental treatments. The mice were injected with different doses of PBS, imipenem, LF, LPO, and LPO and exposed to different treatments. The results showed promising results in treating nosocomial pneumonia.	Studies have demonstrated that camel colostrum LPO and camel colostrum LF have significant antibacterial activity against Acinetobacter baumannii isolates. LPO showed higher activity than LF at the same concentrations. In vivo, a combination of imipenem, LPO, LF, and a crude combination significantly reduced bacteria in lung and blood cultures. LPO and LF also synergistically affected proinflammatory cytokines in treated mice.	[20]
Antiviral	In vitro	The study tested influenza virus susceptibility to LPO products and substrate specificity in a cell-free system. Madin–Darby canine kidney cells were used to evaluate viral inactivation. The system used for virus inactivation included LPO, SCN^−^/I^−^, glucose, and glucose oxidase	All tested influenza strains were inactivated by LPO. LPO did not prefer substrates to inactivate H1N1 and H1N2 viruses. H3N2 strains were inactivated better using iodide than thiocyanate as the LPO substrate.	[21]
Antiviral	In vitro	Milk proteins LF, angiogenin-1, α-lactalbumin, β-lactoglobulin, LPO, casein, lactogenin, and glycolactin inhibited HIV-1 RT, α-glucosidase, β-glucosidase, and β-glucuronidase, with succinylation effect examined using ELISA.	LPO, both unmodified and succinylated, showed significant inhibitory activity on HIV-1 RT. Succinylated LPO showed small inhibitory activity on α-glucosidase and β-glucosidase. Unmodified proteins, except α-lactalbumin and casein, showed significant inhibition. Succinylation increased inhibitory activity of human LF, glycolactin β-lactoglobulin, and α-lactalbumin and casein.	[22]
Antiviral	The study is an evaluation of datas from different sources as a completion	A compilation that includes population, vaccination ratio, infection ratio, and rate of deaths caused by COVID-19 of some countries was prepared.	The study found that Asian countries with high-iodine-containing diets, like Japan, South Korea, and India, had significantly lower COVID-19 death rates compared to western countries, possibly due to differences in iodine intake.	[23]
Antiviral	In vitro	The antiviral activities of human LPO, bovine LPO, and camel LPO against Herpes Simplex Virus Type 1 (HSV-1) were evaluated using cytotoxic effect assay and plaque assay. The viruses were treated with LPO at different concentrations and infected with vero cells.	At concentrations of 0.1, 0.2, 0.3, 0.4, and 0.5 mg/mL, bovine milk LPO had 24, 38, 62, 80, and 100% anti-HSV-1 activity; human colostrum had 10, 16, 30, 44, and 66%; and camel milk had 12, 18, 34, 50, and 70%.	[85]
Antiviral	In vitro	The study investigated the protective and neutralizing effects of bLPO, cLPO, and hLPO on hepatitis C virus (HVC) genotype 4. HepG2 cells were pre-incubated with purified LPO samples and infected with 2% HCV infected serum. The effects of bLPO, cLPO, and hLPO on HCV replication were also examined. LF was used as a control due to its strong activity against HCV G4.	The study revealed that LPO, including bLPO, cLPO, and hLPO, did not protect cells from HCV entry, but they effectively neutralized HCV particles and inhibited entry into HepG2 cells.	[84]
Antifungal	In vitro	LPO-containing systems were investigated for candidacidal action against Candida albicans ATCC strains 18804, 10231, and 11006, comparing CFU and cell viability loss. The study also explored how component concentrations affected candidacidal action.	The system of bovine LPO, GO, glucose, and KSCN showed varying candidacidal activity on different strains. The system’s activity increased with preincubation time, with the highest activity on *Candida albicans* ATCC strain 11006.	[24]
Antifungal	In vitro	The antifungal activity of LPOS was tested on various fungi, using a mixture of H_2_O_2_, KSCN, and goat milk LPO, with concentrations ranging from 30 to 1 mg/mL	Candida albicans and Pythium sp. were resistant to the goat milk LPO-H_2_O_2_-KSCN system. MIC values of goat LPO in the system against the other strains tested were determined in the study.	[81]
Antifungal	In vitro	The anti-candida activities of LF and LPOS were tested using bovine LF, bovine LPO, and Candida albicans. OSCN^−^ solution was produced by using the enzyme system that consisted of 0.16 mg/mL LPO with 7.5 mM NaSCN and 3.75 mM H_2_O_2_ in a buffer	LF and LPOS exhibit significant anti-candida activity on Candida albicans, impacting growth morphology, metabolic activity, and adhesive hyphal form, affecting cellular metabolism	[83]
Antifungal	In vitro	Postharvest mangoes were treated with chitosan coatings with or without LPOS. Two LPOS solutions were used, one of them containing iodine (LPOSI) while the other one did not. Antifungal activity was tested by using strains of *Colletotrichum gloeosporioides*, *Phomopsis* sp. RP257, *Pestalotiopsis* sp., and *Lasiodiplodia Theobromae* ngr 05 A	According to results, there is a synergistic effect of chitosan and LPOS. Inhibitory effect of the coatings was improved by LPOS in all cases. Some strains showed a resistance to chitosan while they were more sensitive to LPOS.	[82]
Antifungal	In vitro and in vivo	A coating made of chitosan solutions and LPOS was produced, tested in vitro and in vivo against fungal strains, with a 5% concentration in the film-forming solution	In vitro results showed that chitosan solutions at 1% and 1.5% without LPOS completely inhibited *Pestalotiopsis* sp. growth, while low inhibition was observed at 0.5%. In vivo results showed increased inhibition, with *Phomopsis* sp. RP257 less sensitive. LPOS with or without iodide increased inhibition at 0.5% chitosan.	[25]
Antioxidant and Therapeutic	In vivo	The study involved 40 adult male albino rats divided into four groups, each with 10 rats, and exposed to experimental treatments to investigate the effects of LPO on aflatoxin B1-induced nephrotoxicity	Measurements indicated that LPO treatment after AFB1 intoxication in group 4 approached all of the values to the values of group 1, which consist of healthy individuals. LPO treatment ameliorated the deteriorations caused by AFB1	[87]
Antitumor and Anti-inflammation	In vivo	Researchers deleted the LPO gene from mice	LPO gene deletion in mice leads to higher rates of diseases like cardiomyopathy, carditis, arteriosclerosis, airway inflammation, glomerulonephritis, digestive system inflammation, and brain pathology. Tumors are also observed in 7 out of 19 one-year-old mice, some of which are overweight or obese	[86]
Anticancer	In vitro	A nanocomposite consisting of graphene oxide (GRO), polyacrylic acid (PAA), Cu, and bovine milk LPO was synthesized and tested against cancer cells.	The findings suggested that the incorporation of LPO with the modified GRO-PAA-Cu composite improved the composite’s selectivity against all treated cancer cells. Furthermore, the results demonstrated that the modified GRO-PAA-Cu-LPO complex enhanced cell cycle arrest in treated cancer cells compared to untreated cells.	[89]
Diagnosis	In vitro and in silico	LPO activity in cow milk was measured to determine mastitis in dairy cows.	LPO activity level can be used as a parameter to determine mammary gland health of cows due to LPO activity increases when mastitis occurs in cows. LPO activity reflects the mammary gland health of cows.	[32]

LPO: lactoperoxidase, LPOS: lactoperoxidase system, LF: lactoferrin, LAB: lactic acid bacteria, XO: xanthine oxidase, GRO: graphene oxide, PAA: polyacrylic acid, GO: glucose oxidase, Glu: α-D-glucose.
